# Novel Insight Into the Role of *ACSL1* Gene in Milk Production Traits in Buffalo

**DOI:** 10.3389/fgene.2022.896910

**Published:** 2022-06-06

**Authors:** Yuxin Lin, Hui Sun, Aftab Shaukat, Tingxian Deng, Hamdy Abdel-Shafy, Zhaoxuan Che, Yang Zhou, Changmin Hu, Huazhao Li, Qipeng Wu, Liguo Yang, Guohua Hua

**Affiliations:** ^1^ Key Lab of Agricultural Animal Genetics, Breeding and Reproduction of Ministry of Education, College of Animal Science and Technology, Huazhong Agricultural University, Wuhan, China; ^2^ Shenzhen Institute of Nutrition and Health, Huazhong Agricultural University, Shenzhen, China; ^3^ Shenzhen Branch, Guangdong Laboratory for Lingnan Modern Agriculture, Genome Analysis Laboratory of the Ministry of Agriculture, Agricultural Genomics Institute at Shenzhen, Chinese Academy of Agricultural Sciences, Shenzhen, China; ^4^ Guangxi Key Laboratory of Buffalo Genetice, Breeding and Reproduxtion, Guangxi Buffalo Research Institute, Chinese Academy of Agricultural Sciences, Guangxi, China; ^5^ Department of Animal Production, Faculty of Agriculture, Cairo University, Giza, Egypt; ^6^ National Center for International Research on Animal Genetics, Breeding and Reproduction (NCIRAGBR); Frontiers Science Center for Animal Breeding and Sustainable Production; Key Laboratory of Smart Farming for Agricultural Animals, Huazhong Agricultural University, Wuhan, China

**Keywords:** buffalo, milk production traits, ACSL1, genetic mutation, mammary gland epithelial cells

## Abstract

Understanding the genetic mechanisms underlying milk production traits contribute to improving the production potential of dairy animals. Long-chain acyl-CoA synthetase 1 (ACSL1) plays a key role in fatty acid metabolism and was highly expressed in the lactating mammary gland epithelial cells (MGECs). The objectives of the present study were to detect the polymorphisms within *ACSL1* in Mediterranean buffalo, the genetic effects of these mutations on milk production traits, and understand the gene regulatory effects on MGECs. A total of twelve SNPs were identified by sequencing, including nine SNPs in the intronic region and three in the exonic region. Association analysis showed that nine SNPs were associated with one or more traits. Two haplotype blocks were identified, and among these haplotypes, the individuals carrying the H2H2 haplotype in block 1 and H5H1 in block 2 were superior to those of other haplotypes in milk production traits. Immunohistological staining of *ACSL1* in buffalo mammary gland tissue indicated its expression and localization in MGECs. Knockdown of *ACSL1* inhibited cell growth, diminished MGEC lipid synthesis and triglyceride secretion, and downregulated *CCND1*, *PPARγ*, and *FABP3* expression. The overexpression of *ACSL1* promoted cell growth, enhanced the triglyceride secretion, and upregulated *CCND1*, *PPARγ*, *SREBP1*, and *FABP3*. *ACSL1* was also involved in milk protein regulation as indicated by the decreased or increased β-casein concentration and *CSN3* expression in the knockdown or overexpression group, respectively. In summary, our present study depicted that *ACSL1* mutations were associated with buffalo milk production performance. This may be related to its positive regulation roles on MGEC growth, milk fat, and milk protein synthesis. The current study showed the potential of the *ACSL1* gene as a candidate for milk production traits and provides a new understanding of the physiological mechanisms underlying milk production regulation.

## Introduction

Water buffalo (*Bubalus bubalis*) is the second-largest milk producer contributing more than 15% of the world’s total milk production. Buffalo is considered the most promising species in developing countries due to its high adaptability to local environmental conditions along with their significant contribution to milk and meat production (Du et al., 2019). Buffalo milk has a higher nutritional value in fat, protein, and iron and less cholesterol content compared to that of dairy cow milk (Barłowska et al., 2011). However, the low milk yield limited buffalo industry progress. Therefore, improving the buffalo milk yield while maintaining its high milk quality is the major challenge for modern buffalo breeding.

Genomic information can be directly utilized through genomic selection (GS) without the knowledge about the biological function of the genetic markers used for prediction, where GS mainly depends on the linkage disequilibrium (LD) among genetic markers and loci associated with the trait variation to create the prediction equation (Goddard et al., 2011; Wang et al., 2019). Recently, it has been reported that incorporating prior physiological knowledge and pre-selected genetic variants into GS increased the accuracy of prediction (Hayes and Daetwyler, 2019). Identification of these genetic loci can be achieved by genome-wide association studies (GWASs) and/or candidate gene approaches. In this issue, several published reports were performed to detect the genomic loci associated with milk production traits in Brazilian, Chinese, Egyptian, Iranian, Italian, and Philippine buffalo (Abdel-Shafy et al., 2020). However, none of these loci was overlapped among different populations and validated, which indicates a modest effect of each SNP and complexity of milk production traits. In this case, candidate gene approaches would be required to accurately identify the genetic markers and causative mutations associated with the relevant trait (Wilkening et al., 2009).

One of the promising candidate genes affecting milk production traits and mammary gland development is the long-chain acyl-CoA synthetases (ACSL), which have been previously detected in dairy cattle (Liang Y. et al., 2020). *ACSL* isoforms (*ACSL1*, *ACSL3*, *ACSL4*, *ACSL5*, and *ACSL6*) differ in various tissues, suggesting that each isoform may have a unique role in a specific tissue (Li et al., 2010). ACSL1 is predominantly expressed in the mammary gland epithelial cells (MGECs) in dairy cattle and is consistently upregulated before the peak of lactation (Bionaz and Loor, 2008). ACSL1 is the most prevalent long-chain acyl-CoA synthetase subtype in major metabolic tissues, which catalyzes fatty acids (FAs) to form acyl-CoA *via* an ATP-dependent process before entry into different intracellular metabolic pathways (Shi et al., 2017). Afterward, it becomes oxidized to provide acylated proteins and complex lipids such as triacylglycerol, phospholipids, and cholesterol esters (Jiang et al., 2019). Considering that triglycerides constitute over 98% of the milk fat composition, it is reasonable to propose that *ACSL1* might regulate triglyceride synthesis and related functions in MGECs.

Therefore, the objectives of this study were to identify genetic mutations of the *ACSL1* gene in buffalo and detect the association between these genetic markers and milk production traits in the tested populations. Furthermore, we tried to explore the regulatory roe of *ASCL1* on MGEC proliferation, lipid distribution, triglycerides, and β-casein synthesis.

## Materials and Methods

### Samples and Phenotypes

A total of 331 buffalo blood DNA samples and relevant milk production records were derived from our previous studies (Deng et al., 2018; Li et al., 2018; Ye et al., 2021). In those previous studies, the Ethical Animal Care and Use Committee of Federico II University of Naples (Italy) approved the experimental design and animal treatment (Deng et al., 2018; Li J. et al., 2020). In addition, all purebred Mediterranean buffalos were selected from four herds in the southern part of Italy, and milk production traits were provided by the Italian Buffalo Breeders Association (ANASB) and the Italian Agricultural Research Council (CAR). Milk production traits were peak milk yield, total milk yield, milk fat yield, milk fat percentage, milk protein yield, and milk protein percentage. All the milk production records were adjusted to 270 days in milk as previously described (Liu J. et al., 2020). The Nanodrop 2000 spectrophotometer (Thermo-Fisher Scientific, Wilmington, DE) and 1.5% agarose gel was used to determine the concentration and quality of extracted DNA.

### SNP Identification and Genotyping

Fifty buffalo samples were randomly selected to identify the variants of the *ACSL1* gene by pooled DNA sequencing. According to the buffalo *ACSL1* genomic sequence (GenBank accession number NC_059157.1); promoter, 3′UTR, 5′UTR, and all the exon sequences were used for selective amplification by the polymerase chain reaction (PCR) (Table 1). The PCR products were detected by agarose gel electrophoresis and sequenced by BGI Biotechnology (Co., Ltd., Shenzhen, China). DNAstar 7.1 software (Co., Inc. Madison, Wisconsin, United States) was used to identify mutations in the sequence. Genotyping was performed by matrix-assisted laser desorption by Compass Biotechnology (Co., Ltd., Beijing, China).

**TABLE 1:** Primer information for the buffalo *ACSL1* gene.

Primer	Sequence (5′-3′)	Region	Start and end position	Product length [bp]
1	F: GTG​GTT​GAA​GGT​GGA​AGA​CAC​GA	promoter	−330–90	339
	R: GGT​CCC​CGA​TGC​TAT​TTA​AGG​G			
2	F: CTC​CTA​GGC​TGC​AGC​GAG​TGG​CTG​GA	5‘UTR	12–250	239
	R: TGG​CCG​GCA​GGG​TAG​CCT​TAG​ATC			
3	F: GTT​TGT​CAC​AGC​ATC​CCT​CCT	exon 1	22,036–22,823	788
	R: AAT​TTG​GGG​ATG​AGC​CTC​TGC			
4	F: GTG​GAG​GAT​TTA​TGT​CAG​ACG​C	exon 2	35,590–35,967	378
	R: AAG​TTA​CAG​GAG​GAG​ATA​GGG​AG			
5	F: AGC​AAA​ACT​CAG​ACC​CAA​ACC	exon 3	36,387–37,069	683
	R: AGC​ACC​CCT​CAA​GAC​AGA​AAG			
6	F: TTT​GTG​TCC​CTG​GAT​TGC​TTT	exon 4	39,708–40,127	420
	R: ATT​CTC​TGT​GCT​TTG​GTT​GCC			
7	F: GGC​AAG​TGT​TTT​GTT​CAT​TAG​G	exon 5 and exon 6	42,551–43,165	615
	R: GTG​TTC​AGG​GAA​GGG​GGC​AGG​G			
8	F: CTT​GGA​ATC​AGT​CCT​GTT​TC	exon 7 and exon 8	45,470–46,200	731
	R: GTC​TTA​GAG​GGT​GCG​TGT​AG			
9	F: AAA​GAC​ATC​AGC​CCT​GGG​ATT​T	exon 9	46,378–47,145	768
	R: TTG​GGG​ATC​AGG​TCC​ATA​GTG			
10	F: TGC​TCT​GAA​ATA​AAT​GGA​GAA​T	exon 10	49,297–49,530	234
	R: TGC​AAG​CGG​TAA​AAA​TGA​AAT​G			
11	F: ACC​GCA​ACT​AGA​GAA​AAG​CC	exon 11	51,069–52,013	945
	R: ATT​GTC​AAG​GTG​AGA​AAA​CG			
12	F: AGT​GGG​GTT​GTT​TCC​TCT​TT	exon 12	53,396–54,116	721
	R: TCT​CGC​TGA​CCT​TCT​CTT​TTA			
13	F: CGG​AAC​CAA​ACC​CGT​CAG​GTG​T	exon 13	54,519–54,860	342
	R: CCG​AAG​AAA​AGA​AGG​GGC​ACA​T			
14	F: TTG​TGG​TAT​TGT​CTT​CTG​TGT​G	exon 14	55,444–55,891	448
	R: CTC​TGA​ACC​TAG​TAT​AAG​GGG​C			
15	F: TTG​TTG​GAG​ATC​AAA​GCA​ATC​T	exon 15	58,616–58,930	315
	R: CAT​GCC​CCC​ACC​CCC​TGA​GAC​T			
16	F: ATA​GAA​CTG​ACC​CCA​GCC​CT	exon 16	59,516–60,154	639
	R: TCA​AAC​CAG​AAG​CAG​CAA​CC			
17	F: CTC​ATC​CCT​TCT​CTG​TCT​CAC​T	exon 17	61,116–61,562	447
	R: CCT​GGA​CGT​CTT​ATA​ATA​TTG​T			
18	F: TAT​CTA​TCC​CAT​TAT​TTG​CG	exon 18 and exon 19	63,025–63,816	792
	R: GAC​AGA​ATC​AGG​ACC​ACA​GC			
19	F: CTC​CCT​ACC​CTA​TGT​TGA​GAT​G	exon 20	63,870–64,229	360
	R: GGT​GGC​TGT​AAG​GCA​GTG​TTC​C			
20	F: TTC​GGA​ATT​ATT​TCA​GGT​CAC​AGA	3‘UTR	63,845–65,550	1706
	R: GCA​ACT​GGA​AGT​GGC​GGG​AT			

F: forward primer. R: reverse primer.

### Linkage Disequilibrium and Association Analysis

Allelic frequencies, genotypic frequencies, polymorphism information content (PIC), and Hardy–Weinberg equilibrium (HWE) were calculated for each locus using PowerMarker Version 3.25. Phased genotypes were partitioned into haplotype blocks using Haploview version 4.2 (Broad Institute, Cambridge, MA, United States). Haploview 4.2 was also used to estimate the LD of all SNPs (Li et al., 2017; Ye et al., 2021). The haplotype structure of each buffalo was inferred by the software Phase 2.1 (Stephens et al., 2001).

The associations between *ACSL1* polymorphisms and milk production traits (peak milk yield, 270 days milk yield, milk fat yield, milk fat percentage, milk protein yield, and milk protein percentage) were analyzed using the custom-made R script (R Core Team., Vienna, Austria), with the following mixed linear model described by Pauciullo et al. (2012):

$$Y_{\mathrm{ijklmn}}=\;\mu +\;G_{i}+P_{j}+S_{k}+F_{l}\;+a_{m\left(i\right)}+e_{\mathrm{ijklmn}},$$

where *Y*_*ijklmn*_ = phenotype observations; μ = overall mean, *G*_*i*_ = the fixed-effect of the i^th^ genotype or haplotype combination; *P*_*j*_ = the fixed-effect of the *j* ^th^ parity (1–7); *S*_*k*_ = the fixed-effect of the k^th^ season (spring is from March to May, summer is from June to August, autumn is from September to November, and winter is from December to January and February of the following year); *F*_*l*_ = the fixed-effect of the l^th^ farm (four different farms); *a*_*m(i)*_ = the random effects of the *m* ^th^ individual buffalo nested within *ACSL1* genotype or haplotype combination i^th^; and *e*_*ijklmn*_ = the random residual. The covariance matrices of random effects of buffalo and residual were assumed to be diagonal $I\sigma_{c}^{2}$ and $I\sigma_{e}^{2}$ , respectively. The least-square means with standard error for multiple comparisons between different genotypes and haplotypes were performed using Bonferroni correction for multiple F-testing.

### Cell Culture and Transfection

The mammary epithelial cell line (MAC-T) was obtained from Bogoo Biotechnology (Co.,Ltd., Shanghai, China). The MAC-T cells were cultured in DMEM/F12 medium (HyClone, United States) containing 10% fetal bovine serum (Gibco, Gaithersburg, MD, United States) and 1% penicillin–streptomycin (HyClone, United States) in a 37°C incubator with 5% CO_2_. MAC-T cells were cultured into six-well plates overnight. Then, MAC-T cells were transfected with *ACSL1* siRNA and NC or pcDNA3.1-*ACSL1* and pcDNA3.1 for 48 or 72 h, using jetPRIME transfection reagent (Polyplus-transfection, FRANCE) following the manufacture’s instruction. The six-well plates were then placed in the incubator and replaced with the fresh complete medium after 6 h.

### Quantitative Real-Time PCR Assay

Total RNA was isolated using TRIzol reagent following the manufacturer’s instructions. The isolated RNA was quantified spectrophotometrically at 260/280 nm and cDNA was synthesized. The qRT-PCR was conducted using Hieff qPCR SYBR Green Master Mix (Yeasen Biotech Co., Ltd, Shanghai, China) to determine the mRNA expression of the target genes using gene-specific primers (Supplementary Table S1). The *GAPDH* was used as a reference gene, and relative expression was measured using the 2^−ΔΔCt^ method.

### Western Blot

The protein was extracted from MAC-T cells, and bicinchoninic acid (BCA) assay was performed to determine the protein concentration. After protein denaturation, 15 μg sample was loaded on SDS-PAGE (10%). Subsequently, the protein was transferred to the PVDF membrane and 5% non-fat milk was used for blocking. The membranes were probed overnight by primary antibodies at 4°C and consequently incubated with secondary antibody (1:5000) for 1.5 h at room temperature. The protein expression was quantified using ImageJ software (National Institutes of Health, Bethesda, MD, United States).

### Cell Viability and Cell Counting Assays

The cells were harvested 72 h after transfection of siRNA or plasmid DNA for the determination of cell viability or cell numbers. The CCK-8 reagent (Dojindo, Japan) was supplemented in each well of the experimental group according to the manufacturer’s instructions, and the cells were incubated for 1 h at 37°C. Next, the cell viability was determined at a wavelength of 450 nm.

### Cell Cycle and Apoptosis Assay

Cell cycle and apoptosis assay was performed based on a protocol established in our laboratory (Wang et al., 2020). Briefly, the cells were pretreated and the cell cycle was detected by using the cell cycle detection kit (KeyGEN BioTECH, Jiangsu, China) according to the manufacturer’s instructions. Apoptosis was analyzed by using the Annexin V-FITC/PI Apoptosis Detection Kit (DOJINDO, Japan). A flow cytometer (BD FACSCalibur, America) was used to detect cell proportions.

### Triglyceride Content Detection and Bodipy Staining

A triglyceride enzyme assay kit (Jiancheng Bioengineering Institute, Nanjing, China) was used to determine the triglyceride contents in the MAC-T cell lysate. The cells were transfected with *ACSL1* siRNA or overexpression plasmid for 72 h, and 100 μl cell lysate was mixed with the working solution. The absorbance was measured at 510 nm using a microplate reader (PerkinElmer Enspire, China).

MAC-T cells were fixed at room temperature using paraformaldehyde (4%) for 15 min, washed twice with PBS, and followed by bodipy staining at room temperature for 10 min and DAPI staining for 5 min in the dark. Then, an anti-fluorescence quenching agent was added, and pictures were taken with an inverted fluorescence microscope (AXIO OBSERVER, ZEISS). Finally, the fluorescence intensity of lipid droplets was measured by ImageJ.

### Enzyme-Linked Immunosorbent Assay

A commercial β-casein kit (Mlbio, Shanghai, China) was used to detect the concentration of β-casein. MAC-T cells were transfected with *ACSL1* siRNA or overexpression plasmid for 72 h and cell culture medium was collected. A total of 50 μl culture medium was used to detect the β-casein secretion level. Absorbance was measured at 450 nm using a microplate reader (PerkinElmer Enspire, China).

### Statistical Analyses for Gene Expression

The statistical analyses of gene functional studies were conducted with SPSS 19.0 software (SPSS Inc., Chicago, IL, United States) and graphing with Graphpad Prism v5.0 (GraphPad Software, Inc., La Jolla, CA, United States). The results are expressed as means ± standard error of the mean (Mean ± SEM). Significant differences between the two groups were compared using Student’s *t*-test, and comparisons among multiple groups were performed with a one-way analysis of variance followed by Dunnett’s test. *P-value* < 0.05 was considered statistically significant. All experiments were conducted at least three times.

## Results

### *ACSL1* Gene SNP Screening and Genotyping

Buffalo DNA pool sequencing data identified a total of twelve potential SNPs in the tested samples (Supplementary Figure S1). Among these twelve SNPs, three were located in the exonic region, and the remaining nine were in the intronic region. The SNP at g.517571A >G, g.524019A >G, g.529284A >G, g.530394C >G, and g.534640A >G was located within intron 9, intron 11, intron 15, intron 16, and intron 20, respectively. The SNPs at g.519961C >T and g.522165C >T were located within intron 10, and the SNPs at g.531913A >C, g.532009C >T, and g.532389A >C were located within intron 17 (Supplementary Table S2). The SNPs at g.492696A >G (exon1), g.492756A >G (exon1), and g.531913A >C (exon17) were all synonymous mutations (Supplementary Table S2).

Detected SNPs were genotyped by MALDI-TOF-MS in 331 Mediterranean buffalo samples (Supplementary Figure S2). Genetic analysis for the tested samples showed that allele frequencies of all SNPs were higher than 15%, and the genotype frequencies of AA in g.492696A >G and g.531913A >C, GG in g.492756A >G and g.534640A >G, TT in g.532009C >T, and CC in g.532389A >C were lower than 10%, and the frequencies of other genotypes were all higher than 10% (Supplementary Table S2). In addition, all the identified SNPs were in accordance with Hardy–Weinberg equilibrium (χ^2^ test, *P* > 0.01) and were moderately polymorphic (0.25 < PIC <0.50) (Supplementary Table S2).

### Association Study of *ACSL1* Genotypes With Milk Production Traits

The association analysis between the twelve detected SNPs and six milk production traits (peak milk yield, 270 days milk yield, milk fat yield, milk fat percentage, milk protein yield, and milk protein percentage) was conducted. The results showed that nine SNPs were associated with at least one of the milk production traits (*p* < 0.05 or *p* < 0.01) (Supplementary Table S3).

The SNPs at g.492696A > G and g.492756A > G loci were significantly associated with milk protein percentage (PP), and buffaloes with mutant type GG at g.492696A > G and g.492756A >G showed the lowest (4.52% ± 0.07%) and highest (4.66% ± 0.09%) PP than those with the A-allele (*p* < 0.05). The SNP at g.517571A >G loci was significantly associated with 270 days of milk yield (MY), and buffaloes with mutant type GG had significantly lower MY (2684.28 ± 89.64 kg) than those with A-allele (*p* < 0.001). The SNP at g.522165C >T loci was significantly associated with MY, milk fat yield (FY), and milk protein yield (PY), where buffaloes with the wild type CC showed the lowest MY(2726.10 ± 87.59 kg), FY(215.38 ± 8.12 kg), and PY(123.93 ± 4.07 kg) compared to the other genotypes (*P* < 0.05 or *P* < 0.01). The SNPs at g.529284A >G loci were significantly associated with FY, where buffaloes with the mutant type GG showed the lowest FY (214.58 ± 8.21 kg) compared to the other genotypes (*p* < 0.05). The SNP at g.531913A >C locus was associated with peak milk yield (PM), MY, and PY, and the heterozygous buffaloes had significantly lower PM (14.82 ± 0.41 kg), MY(2743.01 ± 86.22 kg) and PY(124.68 ± 4.00 kg) than those with homozygous genotypes (*p* < 0.05). The SNPs at g.532009C >T, g.532389A >C and g.534640A >G loci were significantly associated with PM. The buffaloes with the heterozygotic type TC, CA, and GA at g.532009C >T, g.532389A >C, and g.534640A >G, respectively, showed the lowest PM compared to the other genotypes (*p* < 0.01) (Supplementary Table S3).

### Linkage Disequilibrium and Haplotypes Analysis

**FIGURE 1:**
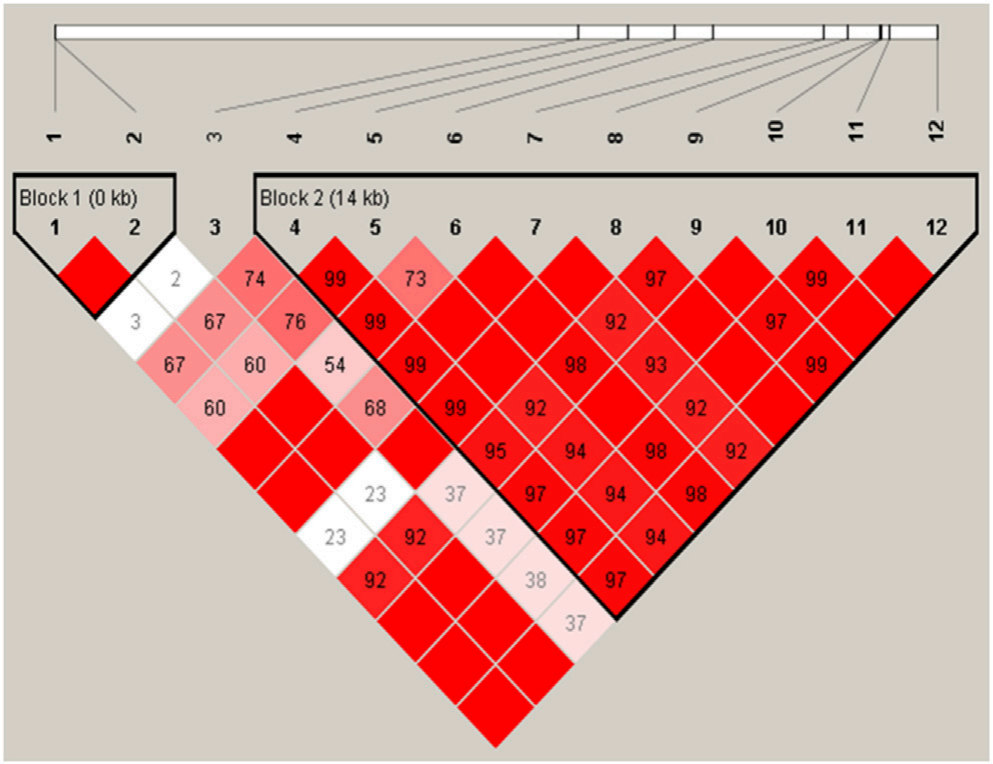
Linkage disequilibrium of the twelve SNPs was detected in the *ACSL1* gene in buffalo. The red squares represent high pairwise linkage disequilibrium, coloring down to white squares of low pairwise linkage disequilibrium, and the linkage disequilibrium is shown as D′.

We further performed LD and haplotype analysis for the twelve detected SNPs, and two haplotype blocks were identified. The LD plot showed that two SNPs were in complete LD resided in haplotype block 1 (D’ = 1), and the remaining nine were in strong LD resided in haplotype block 2 (D' > 0.8) (Figure 1).

We identified three major haplotype combinations (haplotype pairs) (H1H1, H1H2, and H2H2) in block 1 (Table 2), and generated six major haplotype combinations (H1H1, H2H1, H3H1, H2H3, H4H1, and H5H1) in block 2 (Table 3). All of them accounted for a higher frequency of over 5% in the studied subjects. These were selected to perform haplotype-based association analysis. The results showed that all nine haplotype combinations were highly associated with milk protein percentage (*p* < 0.05) (Tables 2, 3). Moreover, the individuals with H2H2 haplotype combination in block 1 (Table 2) and H5H1 in block 2 (Table 3) obtained a higher milk protein percentage than other individuals (*p* < 0.05).

**TABLE 2:** Assocation analysis between haplotypes of block 1 in the *ACSL1* gene and milk production traits.

block1	Frequency (no.)	Sequence	Peak milk yield (kg)	270 days milk yield (kg)	Milk fat yield (kg)	Milk fat percentage (%)	Milk protein yield (kg)	Milk protein percentage (%)
H1H1	0.58 (191)	GA/GA	14.71 ± 0.10	2814.27 ± 22.22	231.37 ± 2.05	8.23 ± 0.04	129.74 ± 0.96	4.63 ± 0.01^b^
H1H2	0.35 (116)	GA/AG	14.62 ± 0.15	2810.01 ± 28.99	228.17 ± 2.63	8.13 ± 0.05	130.98 ± 1.33	4.67 ± 0.02^ab^
H2H2	0.06 (20)	AG/AG	14.70 ± 0.29	2725.47 ± 63.67	221.94 ± 5.80	8.17 ± 0.13	128.10 ± 2.72	4.72 ± 0.04^a^
P value			0.9993	0.5661	0.3419	0.3555	0.4354	0.0149

The values of milk production traits in each genotype are represented as mean ± SE.,. The values with different superscripts within the same column differed significantly at *p* < 0.05.

**TABLE 3:** Assocation analysis between haplotypes of block 2 in the *ACSL1* gene and milk production traits.

block2	Frequency (no.)	Sequence	Peak milk yield (kg)	270 days milk yield (kg)	Milk fat yield (kg)	Milk fat percentage (%)	Milk protein yield (kg)	Milk protein percentage (%)
H1H1	0.2030 (67)	CTG​AGC​CAA/CTG​AGC​CAA	14.89 ± 0.20	2802.20 ± 39.06	224.72 ± 3.66	8.03 ± 0.08	131.05 ± 1.78	4.69 ± 0.02^abc^
H2H1	0.1636 (54)	TCA​GCC​CAA/CTG​AGC​CAA	14.98 ± 0.21	2869.15 ± 38.36	234.24 ± 3.42	8.19 ± 0.08	131.96 ± 1.67	4.61 ± 0.02^c^
H3H1	0.1515 (50)	TCA​GCA​TCG/CTG​AGC​CAA	14.38 ± 0.19	2796.19 ± 42.92	233.22 ± 3.98	8.34 ± 0.08	129.50 ± 1.92	4.64 ± 0.02^bc^
H2H3	0.0758 (25)	TCA​GCC​CAA/TCA​GCA​TCG	14.40 ± 0.26	2650.07 ± 56.45	221.82 ± 5.71	8.33 ± 0.11	123.55 ± 2.57	4.67 ± 0.02^abc^
H4H1	0.0697 (23)	TCG​ACC​CAA/CTG​AGC​CAA	14.54 ± 0.36	2745.29 ± 60.81	229.84 ± 5.77	8.38 ± 0.10	129.79 ± 2.74	4.74 ± 0.04^ab^
H5H1	0.0515 (17)	CTG​ACC​CAA/CTG​AGC​CAA	14.49 ± 0.31	2674.64 ± 68.12	219.56 ± 6.94	8.20 ± 0.14	128.5 ± 3.41	4.81 ± 0.04^a^
P value			0.1446	0.0849	0.2029	0.2611	0.1299	0.0364

The values of milk production traits in each genotypes are represented as mean ± SE.,. The values with different superscripts within the same column differed significantly at *p* < 0.05.

Thus, H2H2 was regarded as a dominant haplotype pair in block 1 and H5H1 was the dominant haplotype pair in block 2 for increasing milk protein percentage.

### Expression and Localization of *ACSL1* in Buffalo Mammary Gland

**FIGURE 2:**
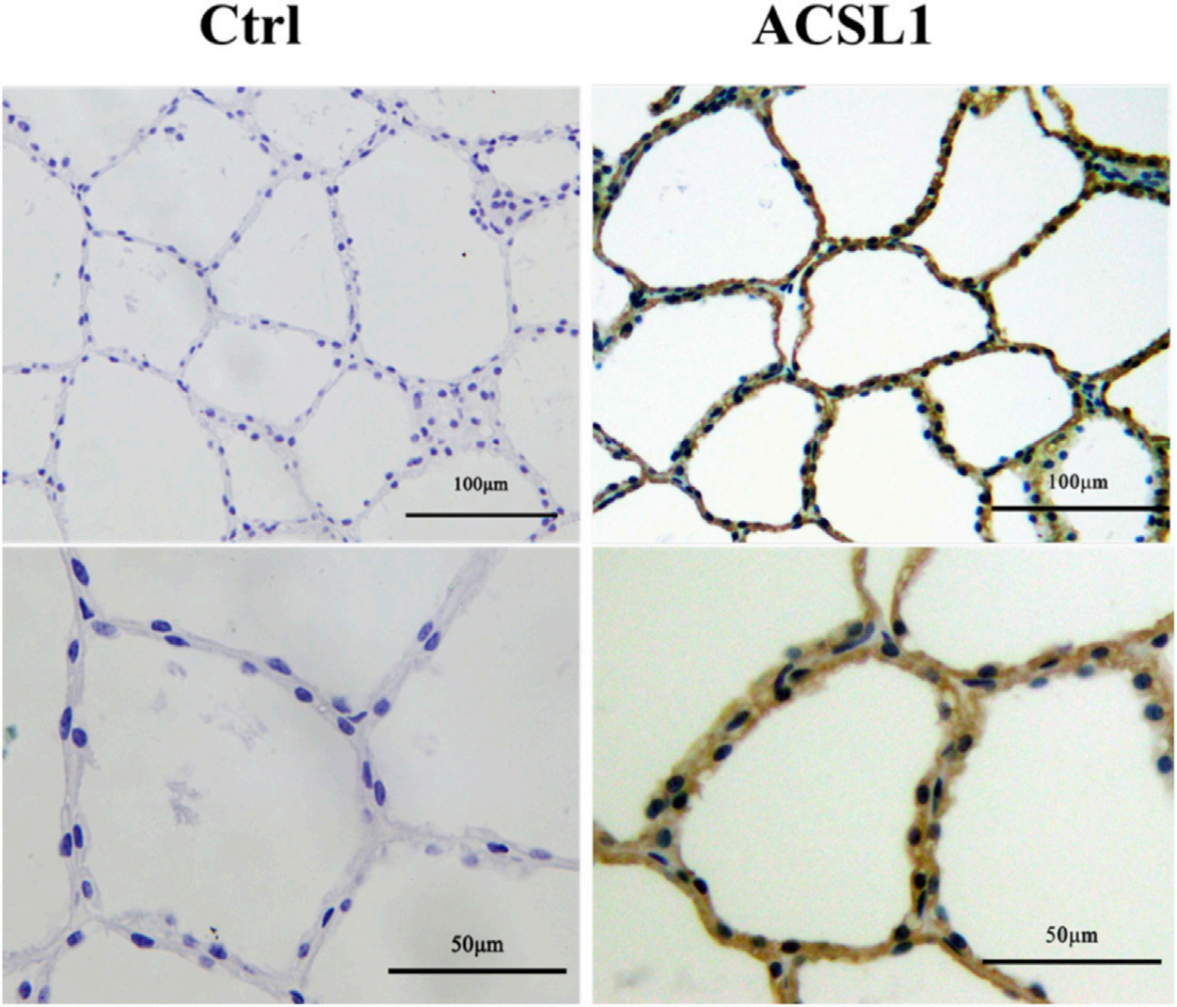
Expression and localization of ACSL1 in buffalo mammary gland. Immunohistochemistry staining of ACSL1 in buffalo mammary gland tissue. The brown color indicated ACSL1 immuno signal, and the nuclei were counterstained in blue. Scale bar: 100 μm (upper panel, 20 times magnification) and 50 μm (lower panel, 40 times magnification).

To identify ACSL1 localization in buffalo mammary gland tissue, we performed immunohistological staining. The result showed that the mammary glands had closely arranged epithelial cells and showed numerous acinar cavities (Figure 2). Furthermore, the buffalo mammary epithelial cells displayed specific immunolabeling for *ACSL1*, of which cytoplasm was intensely labeled (Figure 2).

### *ACSL1* Regulates the Mammary Epithelial Cell Growth

**FIGURE 3:**
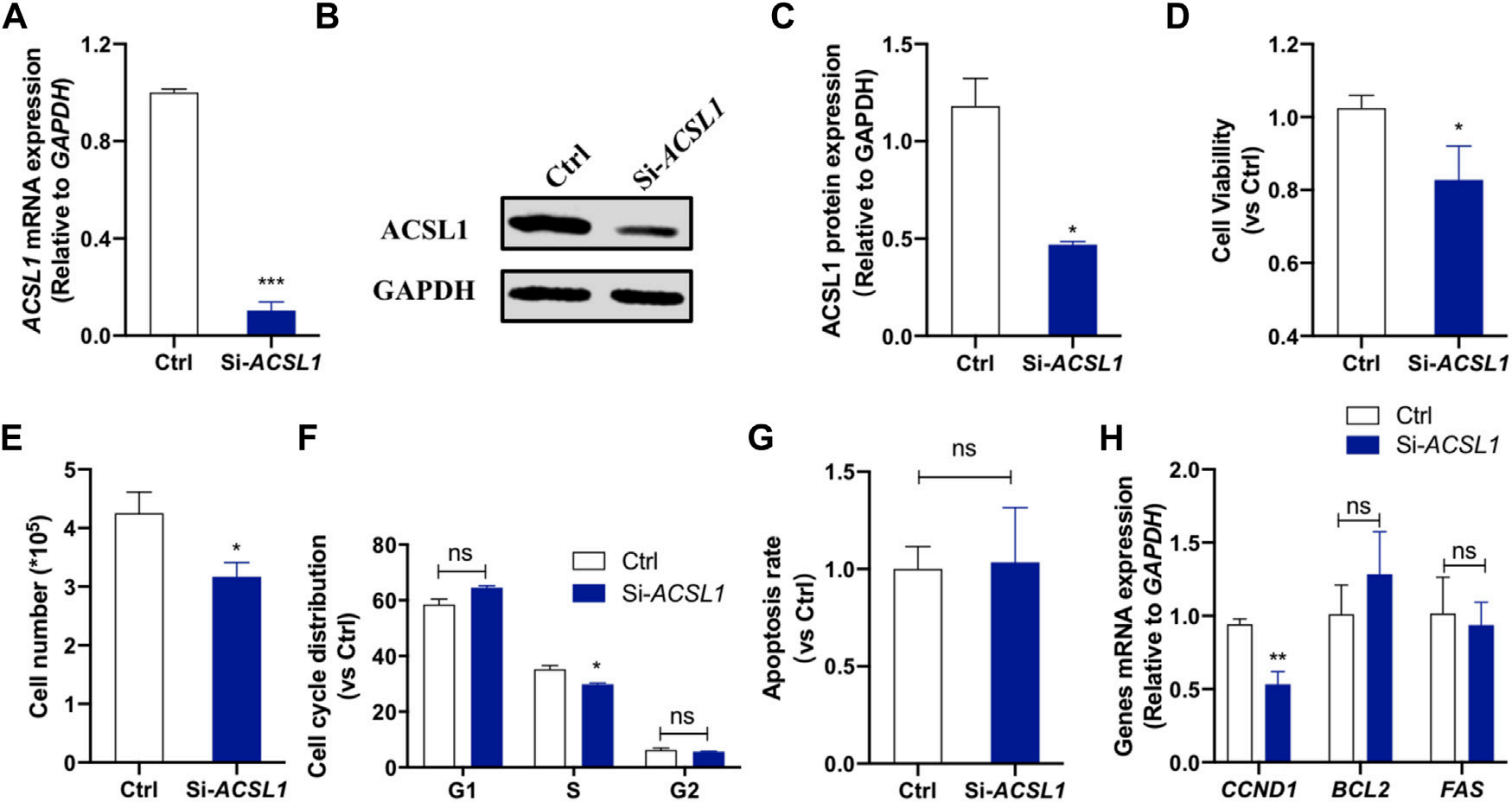
*ACSL1* interference inhibited mammary epithelial cell growth. **(A)** MAC-T cells were transfected with si-*ACSL1*, and fluorescence quantitative qPCR was used to detect the *ACSL1* mRNA levels; **(B,C)** Western Blot detected the protein expression of *ACSL1*; **(D)** CCK-8 assays were applied to check the cell viability after *ACSL1* knockdown; **(E)** Living cell number in control (Ctrl) and *ACSL1* knockdown (Si-*ACSL1*) groups; **(F)** Flow cytometry was used to detect cell cycle progression; **(G)** Quantification of apoptosis by flow cytometry; **(H)** mRNA expression of cell cycle and cell apoptosis–related genes. *GAPDH* was used as the inner control; *p < 0.05, **p < 0.01, and ***p < 0.001, ns: nonsignificant difference.*ACSL1* overexpression was then performed to confirm its regulatory role on mammary epithelial cell growth by using *ACSL1*-overexpressing plasmid (pcDNA3.1-*ACSL1*). Transfection of *ACSL1*-overexpressing plasmid significantly increased *ACSL1* mRNA (Figure 4A) and protein abundance (Figures 4B,C). The CCK-8 assay showed that *ACSL1* overexpression resulted in a significant promotion in cell viability (*p* < 0.01) (Figure 4D). The cell counting test showed that MAC-T cells were significantly increased after *ACSL1* overexpression (*p* < 0.01) (Figure 4E). Then, flow cytometric analysis demonstrated a significant difference in cell cycle distribution in *ACSL1* overexpression cells (*p* > 0.05) (Figure 4F). The cell apoptosis rate showed no significant differences between the control and *ACSL1* overexpression cells (Figure 4G). The overexpression of *ACSL1* upregulated *CCND1* expression (*p* < 0.01), while that of *BCL2* and *FAS* remained unchanged (Figure 4H).

The potential effects of *ACSL1* on cellular functions were investigated in an *in vitro* model using MAC-T mammary epithelial cells. The cells were transfected with siRNA, and knockdown was confirmed by qRT-PCR and Western-blot. The results showed that RNA interference downregulated *ACSL1* mRNA expression by 90% (*p* < 0.001) (Figure 3A) and protein expression by 51% compared to the control group (*p* < 0.05) (Figures 3B,C). To confirm the *ACSL1* role in cell proliferation, we performed CCK-8 assays to examine the effect of *ACSL1* on the viability of MAC-T cells. The results demonstrated that cell viability was significantly reduced by *ACSL1* knockdown (*p* < 0.05) (Figure 3D). In addition, the cell counts were measured with an automatic cell counter, and the results revealed a significant decrease in the number of cells in *ACSL1* knockdown cells (Figure 3E). We next examined cell cycle and apoptosis using flow cytometry. *ACSL1* knockdown resulted in a severe S-phase arrest (*p* < 0.05) (Figure 3F), whereas knockdown of *ACSL1* had no major impact on cell apoptosis progression (*p* > 0.05) (Figure 3G). Consistently, *ACSL1* knockdown inhibits the cell cycle–related gene (*CCND1*) expression (*p* < 0.01), without changing *Bcl2* and *FAS* expression (Figure 3H).

**FIGURE 4:**
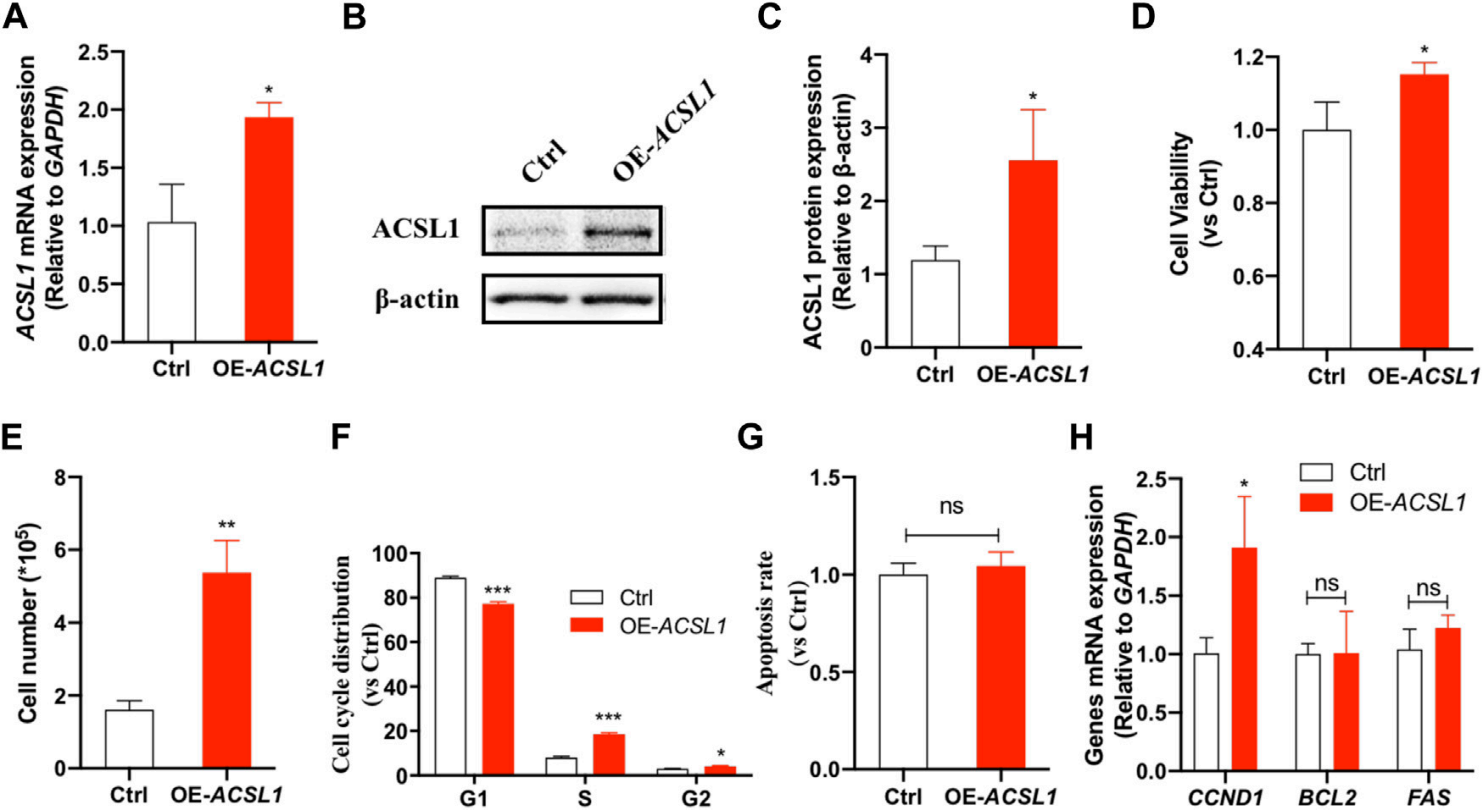
*ACSL1* overexpression promotes cell growth. **(A)** MAC-T cells were transfected with pcDNA3.1-*ACSL1* for 48°h, and fluorescence quantitative qPCR was used to detect the changes of *ACSL1* mRNA levels; **(B,C)** Western blot detects the protein expression of ACSL1; **(D)** CCK-8 assays were applied to check the cell viability after *ACSL1* overexpression; **(E)** Living cell number in control (Ctrl) and *ACSL1* overexpression (OE-*ACSL1*) groups; **(F)** Flow cytometry was applied to cell cycle progression; **(G)** Quantification of apoptosis by flow cytometry; **(H)** mRNA expression of cell cycle and cell apoptosis–related genes. *GAPDH* was used as inner control; *p < 0.05, **p < 0.01, and ***p < 0.001, ns: nonsignificant difference.

### *ACSL1* Regulate Mammary Epithelial Cell Lipogenesis

**FIGURE 5:**
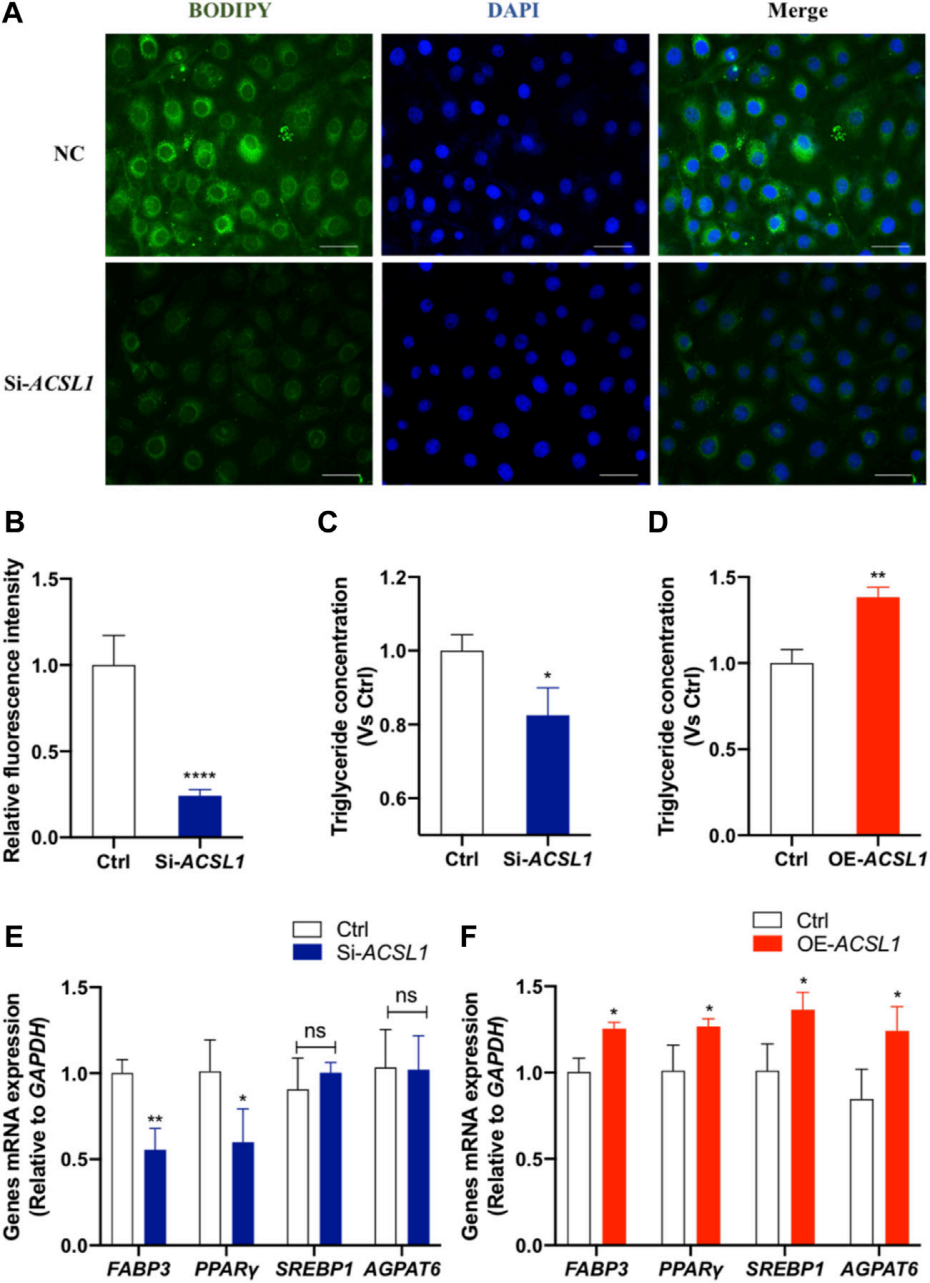
*ACSL1* regulated lipogenesis and triglyceride synthesis in MAC-T cells. **(A)** MAC-T cells were transfected either with *ACSL1* siRNA or negative control for 72 h. Bodipy staining (green) was used to indicate the lipid distribution, and nuclei were stained by DAPI (blue). Scale bar: 20 µm; **(B)** Quantification of *BODIPY* + fluorescent signal density; **(C,D)** Triglyceride concentration was detected in the cell lysate. Triglyceride concentration was normalized by control (Ctrl); **(E,F)** mRNA expression of lipid metabolism–related genes after *ACSL1* knockdown or overexpression, and *GAPDH* was used as the inner control. *p < 0.05, **p < 0.01, and ***p < 0.0001, ns: nonsignificant difference.

To make a thorough exploration of *ACSL1* function in mammary epithelial cells, we detected the effect of *ACSL1* on milk fat synthesis. The BODIPY staining of neutral lipid accumulation confirms the reduction of lipid droplets in *ACSL1* knockdown cells (*p* < 0.001) (Figures 5A,B). The secretory effect of *ACSL1* on the level of triglyceride (TG), a major lipid milk fat, was examined. The results showed that *ACSL1* knockdown reduced the secretion of triglycerides (*p* < 0.05) (Figure 5C) and the overexpression of *ACSL1* led to an increase of triglyceride content of 38% over the control group (*p* < 0.01) (Figure 5D) in MAC-T cells. We next examined the expression of genes associated with lipid anabolism. The results showed that *ACSL1* knockdown decreased *FABP3* (*p* < 0.01) and *PPARγ* (*p* < 0.05) expression but did not alter the mRNA level of *SREBP1* and *AGPAT6* (*p* > 0.05) (Figure 5E). In contrast, the overexpression of *ACSL1* promotes the expression of *FABP3*, *PPARγ*, *SREBP1*, and *AGPAT6* (*p* < 0.05) (Figure 5F).

### *ACSL1* Regulated Mammary Epithelial Cell Casein Synthesis

**FIGURE 6:**
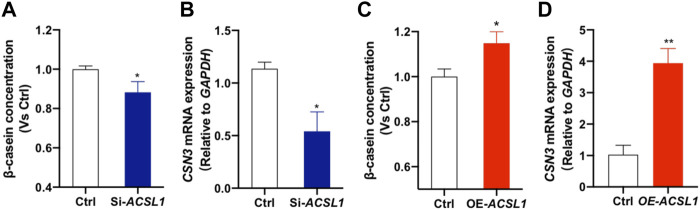
*ACSL1* regulated β-casein synthesis and κ-casein expression in MAC-T cells. **(A,C)** Content of β-casein in the MAC-T cell culture supernatant was determined by ELISA, and β-casein concentration was normalized by control (Ctrl); **(B–D)** mRNA expression of κ-casein (*CSN3*) after *ACSL1* knockdown or overexpression, and *GAPDH* was used as the inner control. *p < 0.05; **p < 0.01.

Our previous association study revealed that *ACSL1* mutation affected the milk protein percentage (Supplementary Tables S4, S5). We further performed the ELISA assay to detect the β-casein (a major lactoprotein) levels in the culture medium and κ-casein (*CSN3*) expression of mammary gland epithelial cells after *ACSL1* silencing or overexpression. Here, we showed that *ACSL1* knockdown significantly attenuated β-casein production and downregulated κ-casein (*CSN3*) expression (Figures 6A,B, *P* < 0.05). The overexpression of *ACSL1* significantly increased β-casein production and *CSN3* expression (*p* < 0.05 or *p* < 0.01) ().

## Discussion

Milk production traits are complex in nature, where several genes are involved in their regulation along with different environmental factors. Nowadays, the selection of superior animals for increasing the frequency of desired alleles with a positive effect on a given trait focuses on genetic improvement in livestock. Moreover, identifying the single-nucleotide polymorphisms (SNPs) for milk production traits is currently being essential to increase the accuracy of prediction for animal genetic merit, which is useful for genetic improvement of production traits in livestock (Jiang et al., 2019). Importantly, the *ACSL1* is deregulated in many tumors, leading to abnormal lipid synthesis and extracellular lipid uptake that promotes uncontrolled cancer cell proliferation (Rossi Sebastiano and Konstantinidou, 2019). Recently, a lot of evidence indicated that mutation in the *ACSL1* might affect the production performance. Quantitative trait loci analysis demonstrated that *ACSL1* is a candidate gene for the location and function of the fatty acid composition of bovine skeletal muscle (Widmann et al., 2011). Polymorphism analysis of *ACSL1* suggested an association between genotype and backfat thickness. Manichaikul et al. (2016) demonstrated that three SNPs located in the intronic regions of *ACSL1* are associated with the level of glucose in fasting or diabetes. However, *ACSL1* genetic data regarding milk production are very preliminary, especially in buffalo. In this context, Liang S. S. et al. (2020) have observed high expression levels of the *ACSL1* mRNA in the mammary tissue of lactating buffalo, suggesting that *ACSL1* may be related to lactation performance of buffalo. In our study, the presence of ACSL1 protein was detected in mammary epithelial cells by immunohistochemistry, and it was mainly in the cytoplasm, which was consistent with the findings of Wang et al. (2017) in the study on the relationship between *ACSL1* and human breast cancer.

The haplotype analysis of the *ACSL1* promoter region in *Bos grunniens* has a significant correlation with the milk protein percentage and milk fat percentage. In the present study, identified *ACSL1* polymorphisms (g.531913A >C, g.532009C >T, g.532389A >C, and g.534640A >G) were associated with peak milk yield (g.517571A >G, g.522165C >T, and g.531913A >C), with 270 days milk yield (g.522165C >T and g.529284A >G), with milk fat yield (g.5522165C >T and g.531913A >C), with milk protein yield (g.492696A >G and g.492756A >G), and with milk protein percentage. Li et al. (2012) identified four SNPs in the pig *ACSL1*, and the mutations of exon were all synonymous. In our study, the SNPs of g.492696A >G, g.492756A >G, and g.531913A >C located in the exon were synonymous substitutions. Apparently, the synonymous mutations do not alter the amino acid encoded by the affected codon due to the degeneracy of the genetic code but change the DNA and RNA sequence (Sharma et al., 2019). Nonetheless, recent studies suggested their significant impact on splicing, RNA stability, RNA folding, translation, or co-translational protein folding. In addition, many studies have revealed that synonymous mutations play a role in a variety of human diseases and can be linked to a patient’s clinical outcome or responsiveness to treatment (Schutz et al., 2013). Thus, the expression of *ACSL1* may be affected by SNP g.492696A >G (exon1), g.492756A >G (exon1), and g.531913A >C (exon17), which has an influence on milk fat metabolism and ultimately affects some buffalo milk production traits. The remaining nine SNPs found in the intronic region were non-functional SNPs and did not lead to alterations in amino acids. Nevertheless, an increasing amount of evidence reveals that noncoding regions in the genome cause abnormal splicing of gene transcripts. Similarly, Rose (2008) investigated that one is often overlooked. Still, many genes with an intact promoter were essentially not expressed at all without an intron, while many genes with an intact promoter were essentially not expressed at all without an intron. Hence, the SNP of g.517571A >G, g.519961C >T, g.522165C >T, g.524019A >G, g.529284A >G, g.530394C >G, g.532009C >T, g.532389A >C, and g.534640A >G may affect the milk producing traits by affecting ACSL1 protein formation or linkage with other marker loci associated with milk-production traits. As a matter of fact, compared to individual SNPs, LD and haplotypes had more genetic information. Testing multiple SNPs simultaneously can capture the underlying architecture of complex quantitative traits better (Abdel-Shafy et al., 2014). For this purpose, Li et al. (2019) claimed that H2H3 and H2H2 in Chinese Holstein cow *FBP2* were the dominant haplotype combinations, improving milk yield, milk fat, and milk protein. Our study indicated that *ACSL1* functional diplotypes (H1H1, H1H2, and H2H2) in block 1, comprising haplotypes from two detected SNPs (g.492696A >G and g.492756A >G), and (H12H12, H1H12, H4H12, H1H4, and H5H12) in block 2, comprising haplotypes from nine detected SNPs (g.519961C >T, g.522165C >T, g.524019A >G, g.529284A >G, g.530394C >G, g.531913A >C, g.532009C >T, g.532389A >C, and g.534640A >G), were associated with milk protein percentage. Under selection, haplotype-based approaches have further advantages, suggesting that H5H1 diplotypes in block 2 were selected during artificial selection. This research is the first study to examine *ACSL1* polymorphisms associated with buffalo milk production traits to the best of our knowledge. The exploration of *ACSL1* genetic variants can provide added value to buffalo molecular breeding.

The quantity and activity of mammary epithelial cells are known to be linked with lactation and play a key role in the growth of mammary glands (Boutinaud et al., 2004). According to the NCBI buffalo and dairy cow *ACSL1* genomic sequences (Gene ID: 102414095 and Gene ID: 537161, respectively), buffalo *ACSL1* showed close homology (98%) to dairy cow *ACSL1* sequences. Therefore, we explored the *ACSL1* regulation on mammary epithelial cell growth. In all types of cell cultures, the measurement of cell viability is crucial and is often used to determine cell proliferation within a cell population. Chen et al. (2016) found that *ACSL1* knockdown inhibited breast cancer cell proliferation. In the present study, *ACSL1* knockdown reduced cell viability, and *ACSL1* overexpression significantly increased cell viability, indicating that the effect of *ACSL1* on cell viability was consistent with the aforementioned research results. The majority of the mammary epithelial cells are secretory cells that undergo functional differentiation to generate milk during pregnancy (Qiu et al., 2019). The number of mammary epithelial cells affect milk yield during lactation. The decline in milk production after peak lactation is accompanied by a gradual reduction in the number of mammary epithelial cells (Boutinaud et al., 2004). In our study, *ACSL1* overexpression significantly increased the cell population, while its depletion downregulated the cell population. These findings were consistent with those of the cell viability analysis, which further supported that *ACSL1* might promote mammary epithelial cell proliferation.

As for cell cycle analysis, Ma et al. (2021) found that *ACSL1* knockdown blocked the cell cycle and stopped prostate cancer cells from proliferating and migrating. Similarly, our results showed that *ACSL1* knockdown resulted in the G1/S-phase arrest and affected the DNA synthesis in mammary epithelial cells, while *ACSL1* overexpression increased the S-phase rate. The cell cycle is a complex process tightly regulated by the cyclins and their catalytic moieties. It has been shown that recombinant complexes of *CDK4* or *CDK6* and *CCND1* are necessary for the G1/S transition (Ma et al., 2015). In our study, we found that *ACSL1* positively regulated *CCND1* expression, which further supported that *ACSL1* regulates the cell cycle and affects cell proliferation. Actually, following a cyclic pattern of lactation–involution–lactation, mammary epithelial cells experience multiplication, differentiation, apoptosis, and regeneration (Monks et al., 2008). As for apoptosis analysis, Zhao et al. (2019) concluded that a lack of *ACSL1* causes a generalized impairment in muscle fuel metabolism, which leads to an increase in protein catabolism, resulting in myocyte apoptosis. In addition, inhibition of *ACSL1* during fatty acid loading results in macrophage apoptosis *via* the accumulation of free fatty acids (Pan et al., 2007). Together, these results suggested that *ACSL1* regulated mammary epithelial cell growth and may pose a positive role in bovine milk yield.

The lipids are a major energetic constituent of milk, and the principal lipids of milk are triacylglycerides, representing up to 98% of the total lipids (Liu Z. et al., 2020). Several studies have confirmed that the triglyceride increased after *ACSL1* overexpression or decreased after *ACSL1* knockdown (Li T. et al., 2020). As expected, in our study, *ACSL1* overexpression increased triglyceride in MAC-T cells, and *ACSL1* knockdown decreased triglyceride levels. Lipid droplets, which promote coordination and communication between diverse organelles and serve as key hubs of cellular metabolism, are the most common storage form for neutral lipids. Lian et al. (2016) indicated that bta-miR-181a negatively regulated *ACSL1* and then proved that *ACSL1* positively regulated lipid droplet and triglyceride synthesis. Zhao et al. (2020) also reported that *ACSL1* overexpression resulted in lipid droplet aggregation. Coincidentally, in this study, *ACSL1* knockdown inhibits the accumulation of lipid droplets, which was consistent with the abovementioned results. PPARγ can maintain mature adipocytes and promote adipogenesis. PPARγ activation increased triglyceride content and elevated the number and size of lipid droplets in the mouse liver (Liu et al., 2021). Zhao et al. (2020) found that *ACSL1* overexpression significantly increased *PPARγ* expression and triglyceride secretion, while significantly decreasing FA oxidation–related gene *CPT1A* expression. The results of the present study were consistent with these findings as *ACSL1* positively regulates *PPARγ* expression and triglyceride secretion. However, other studies had dictated that *PPAR* is involved in the β-oxidation of fatty acids in the liver. Li T. et al. (2020) indicated that *ACSL1* negatively regulated *PPARγ* in human liver cells, and *ACSL1* overexpression reduces fatty acid β-oxidation *via* the PPAR*γ* pathway, resulting in a rise in triglyceride levels. In contrast to adipocytes and liver cells, *ACSL1*-deficient macrophages do not reduce β-oxidation (Rubinow et al., 2013). These findings suggested that the function of *ACSL1* may differ in cells. The changes in the upstream signaling cascade and transcriptional networks that regulate *ACSL1* expression, in particular, may have an impact on the entry of fatty acyl-CoAs into several metabolic processes. In addition, *SREBP1* is the key positive regulator in milk fat synthesis of dairy cow mammary epithelial cells (He et al., 2020). *AGPAT6* is highly expressed in mammary epithelium tissue, which is crucial for producing milk fat. *FABP3* upregulated the expression of *SREBP1* and *PPARγ* to increase lipid droplet accumulation. Bionaz and Loor (2008) reported that *ACSL1*, *FABP3*, and *AGPAT6* coordinate and regulate the channeling of fatty acids toward copious milk fat synthesis in bovine mammary glands. In our study, *ACSL1* positively regulated *SREBP1*, *FABP3*, *PPARγ*, and *AGPAT6* mRNA expression. Therefore, it is suggested that the lipogenesis process was regulated by *ACSL1* in MAC-T cells.

Caseins are an important group of proteins in milk that accounted for approximately 80% of milk proteins and are secreted by the mammary epithelial cells (Cavaletto et al., 2008). There are four types of casein: α_s1_-casein, α_s2_-casein, β-casein, and κ-casein, all of which possess different structures and functionality, and both α_s1_-casein and β-casein are major caseins (Cosenza et al., 2021). Wang et al. (2014) found that *Pten* downregulates dairy cow mammary epithelial cell secretion of β-casein. The present study found that *ACSL1* knockdown resulted in a significant reduction in β-casein content, and *ACSL1* overexpression significantly increased β-casein secretion. *ACSL1* polymorphisms were significantly associated with milk protein yield and milk protein percentage. Accordingly, *ACSL1* may affect milk protein synthesis and lactation in MAC-T cells.

## Conclusion

In conclusion, it is demonstrated that twelve SNPs regulate *ACSL1* in buffalo. Four SNPs were significantly associated with peak milk yield; three SNPs were significantly associated with 270 days milk yield; two SNPs were significantly associated with 270 days milk fat yield; two SNPs were significantly associated with 270 days milk protein yield, and two SNPs were significantly associated with milk protein percentage. Three diplotypes in block 1 and six diplotypes in block 2 were associated with protein percentage, and H5H1 in block 2 was the dominant diplotype. Furthermore, *ACSL1* positively regulated the cell growth, triglyceride and casein synthesis, and related gene expressions such as *CCND1* and *PPARγ*. These findings provide evidence that the buffalo *ACSL1* gene may be a potential candidate gene for marker-assisted selection in the buffalo breeding program.

## Data Availability

The datasets presented in this study can be found in online repositories. The name of the repository and link to the data can be found below: Harvard Dataverse; https://doi.org/10.7910/DVN/KBVOI6.

## Abbreviations

ACSL1, long-chain acyl-CoA synthetases; GS, genomic selection; LD, linkage disequilibrium; GWAS, genome-wide association studies; PM, peak milk yield; MY, milk yield; FY, milk fat yield; FP, milk fat percentage; PM, milk protein yield; PP, milk protein percentage; MGECs, mammary gland epithelial cells; TG, triglyceride; PIC, polymorphism information content; HWE, Hardy–Weinberg equilibrium.
