# Supplementary material for: Novel Insight Into the Role of ACSL1 Gene in Milk Production Traits in Buffalo
Source: Front Genet. 2022 Jun 6;13:896910. doi: 10.3389/fgene.2022.896910 (PMC9207818; doi:10.3389/fgene.2022.896910)
Supplement: Supplementary file 1 [file Table1.docx]

**Supplementary Table 1.** Primer sequences used for qRT-PCR assays

| gene | primer sequence(5′-3′) |
| --- | --- |
| *ACSL1* | F: GTGGGCTCCTTTGAAGAACTGT |
|  | R: ATAGATGCCTTTGACCTGTTCAAAT |
| *CCND1* | F: CCGTCCATGCGGAAGATC |
|  | R: CAGGAAGCGGTCCAGGTAG |
| *FABP3* | F: GAACTCGACTCCCAGCTTGAA |
|  | R: AAGCCTACCACAATCATCGAAG |
| *PPARγ* | F: TCAAAGTGGAGCCTGTATC |
|  | R: CATAGTGGAACCCTGACG |
| *GAPDH* | F: CACGTGGAGGGGCCGGACTCATC |
|  | R: TAAAGACCTCTATGCCAACACAGT |
| *SREBP1* | F: CTGACGACCGTGAAAACAGA |
|  | R: AGACGGCAGATTTATTCAACTT |
| *AGPAT6* | F: AAGCAAGTTGCCCATCCTCA |
|  | R: AAACTGTGGCTCCAATTTCGA |
| *BCL2* | F: CTGGGACGCCTTTGTGGAG |
|  | R: GGCCTGTGGGCTTCACTTAT |
| *FAS* | F: CCACGGCTGTCGGTAAT |
|  | R: CGCTCCCACTCATCCTG |

F: Forward primer. R: reverse primer.

**Supplementary Table 2.** Genotype, allele frequencies and genetic diversity parameters of identified SNPs in *ACSL1* gene

| SNPs | location | genotype | genotype number | genotype frequency | alleles | allele frequency | PIC |
| --- | --- | --- | --- | --- | --- | --- | --- |
|  |  |  |  |  |  |  |  |
| g.492696A>G | exon1  22175 | AA | 20 | 0.06 | A | 0.24 | 0.296 |
|  |  | AG | 117 | 0.35 | G | 0.76 |  |
|  |  | GG | 194 | 0.59 |  |  |  |
| g.492756A>G | exon1  22235 | AA | 193 | 0.58 | A | 0.76 | 0.297 |
|  |  | AG | 117 | 0.35 | G | 0.24 |  |
|  |  | GG | 20 | 0.06 |  |  |  |
| g.517571A>G | intron9  47050 | AA | 115 | 0.35 | A | 0.61 | 0.363 |
|  |  | AG | 173 | 0.52 | G | 0.39 |  |
|  |  | GG | 43 | 0.13 |  |  |  |
| g.519961C>T | intron10  49440 | CC | 89 | 0.27 | C | 0.52 | 0.375 |
|  |  | TC | 163 | 0.49 | T | 0.48 |  |
|  |  | TT | 79 | 0.24 |  |  |  |
| g.522165C>T | intron10  51644 | CC | 61 | 0.18 | C | 0.42 | 0.369 |
|  |  | TC | 159 | 0.48 | T | 0.58 |  |
|  |  | TT | 111 | 0.34 |  |  |  |
| g.524019A>G | intron11  53498 | AA | 65 | 0.2 | A | 0.42 | 0.368 |
|  |  | AG | 146 | 0.44 | G | 0.58 |  |
|  |  | GG | 120 | 0.36 |  |  |  |
| g.529284A>G | intron15  58763 | AA | 143 | 0.42 | A | 0.64 | 0.354 |
|  |  | AG | 146 | 0.43 | G | 0.36 |  |
|  |  | GG | 48 | 0.14 |  |  |  |
| g.530394C>G | intron16  59873bp | CC | 99 | 0.3 | C | 0.55 | 0.373 |
|  |  | CG | 164 | 0.5 | G | 0.45 |  |
|  |  | GG | 67 | 0.2 |  |  |  |
| g.531913A>C | exon17  61392 | AA | 13 | 0.04 | A | 0.18 | 0.251 |
|  |  | CA | 93 | 0.28 | C | 0.82 |  |
|  |  | CC | 225 | 0.68 |  |  |  |
| g.532009C>T | intron17  61488 | CC | 225 | 0.68 | C | 0.82 | 0.25 |
|  |  | TC | 94 | 0.28 | T | 0.18 |  |
|  |  | TT | 12 | 0.04 |  |  |  |
| g.532389A>C | intron17  61868 | AA | 223 | 0.67 | A | 0.82 | 0.251 |
|  |  | CA | 97 | 0.29 | C | 0.18 |  |
|  |  | CC | 11 | 0.03 |  |  |  |
| g.534640A>G | intron20  64119 | AA | 223 | 0.67 | A | 0.82 | 0.253 |
|  |  | GA | 96 | 0.29 | G | 0.18 |  |
|  |  | GG | 12 | 0.04 |  |  |  |

**Supplementary Table 3.** Association analysis between detected genotypes of *ACSL1* and milk production traits in Buffalo

| SNPs | genotypes  (no.) | peak milk yield (kg) | 270d milk yield (kg) | milk fat yield (kg) | milk fat percentage (%) | milk protein yield (kg) | milk protein percentage (%) |
| --- | --- | --- | --- | --- | --- | --- | --- |
| g.492696A>G | AA (20) | 15.44±0.53 | 2782.95±108.80 | 220.31±10.03 | 7.97±0.25 | 129.09±5.05 | 4.66±0.09^a^ |
|  | AG (117) | 15.35±0.43 | 2845.17±89.07 | 227.34±8.21 | 7.96±0.21 | 130.72±4.14 | 4.58±0.07^a^ |
|  | GG (194) | 15.21±0.41 | 2799.27±85.17 | 219.85±7.85 | 7.80±0.20 | 127.13±3.95 | 4.52±0.07^b^ |
|  | P value | 0.9755 | 0.4890 | 0. 2705 | 0.3577 | 0.3550 | 0.0212 |
| g.492756A>G | AA (193) | 15.20±0.41 | 2797.55±85.26 | 219.72±7.87 | 7.80±0.20 | 127.08±3.96 | 4.52±0.07^b^ |
|  | AG (117) | 15.37±0.43 | 2846.25±88.74 | 227.03±8.19 | 7.95±0.21 | 130.53±4.12 | 4.57±0.07^b^ |
|  | GG (20) | 15.44±0.53 | 2780.80±108.76 | 220.01±10.03 | 7.96±0.26 | 128.95±5.05 | 4.66±0.09^a^ |
|  | P value | 0.9485 | 0.4499 | 0. 2529 | 0.3849 | 0.3550 | 0.0266 |
| g.517571A>G | AA (115) | 15.43±0.43 | 2868.95±89.43^a^ | 222.09±8.29 | 7.66±0.21 | 130.42±4.16 | 4.50±0.08 |
|  | AG (173) | 15.46±0.42 | 2882.93±86.48^a^ | 227.73±8.02 | 7.90±0.20 | 131.24±4.03 | 4.56±0.07 |
|  | GG (43) | 14.89±0.44 | 2684.28±89.64^b^ | 213.23±8.31 | 7.85±0.21 | 122.63±4.17 | 4.53±0.08 |
|  | P value | 0.3135 | 0.0338 | 0. 0705 | 0.3000 | 0.0696 | 0.7403 |
| g.519961C>T | CC (89) | 15.73±0.45 | 2898.35±92.91 | 227.19±8.60 | 7.81±0.22 | 133.38±4.31 | 4.60±0.08 |
|  | TC (163) | 15.31±0.42 | 2838.20±87.57 | 224.15±8.10 | 7.84±0.21 | 129.65±4.06 | 4.54±0.07 |
|  | TT (79) | 15.16±0.42 | 2779.94±86.23 | 219.23±8.00 | 7.84±0.20 | 126.32±4.00 | 4.53±0.07 |
|  | P value | 0.2952 | 0.1643 | 0. 1419 | 0.6658 | 0.0748 | 0.3304 |
| g.522165C>T | CC (61) | 15.00±0.42 | 2726.10±87.59^b^ | 215.38±8.12^b^ | 7.84±0.21 | 123.93±4.07^b^ | 4.53±0.07 |
|  | TC (159) | 15.36±0.42 | 2862.62±86.69^a^ | 226.03±8.04^a^ | 7.86±0.20 | 130.56±4.03^a^ | 4.55±0.07 |
|  | TT (111) | 15.71±0.44 | 2900.50±90.16^a^ | 226.83±8.36^a^ | 7.76±0.21 | 132.77±4.19^a^ | 4.56±0.08 |
|  | P value | 0.1367 | 0.0326 | 0. 0278 | 0.5155 | 0.0204 | 0.5675 |
| g.524019A>G | AA (65) | 15.16±0.42 | 2786.04±86.75 | 219.15±8.01 | 7.81±0.20 | 126.37±4.02 | 4.52±0.07^b^ |
|  | AG (146) | 15.14±0.43 | 2812.10±89.08 | 221.87±8.23 | 7.83±0.21 | 127.81±4.13 | 4.51±0.07^b^ |
|  | GG (120) | 15.56±0.43 | 2858.40±89.79 | 226.76±8.29 | 7.91±0.21 | 131.85±4.16 | 4.61±0.08^a^ |
|  | P value | 0.5248 | 0.4758 | 0. 1987 | 0.3659 | 0.1789 | 0.0627 |
| g.529284A>G | AA (143) | 15.58±0.43 | 2869.48±88.24 | 226.91±8.16^a^ | 7.87±0.21 | 131.78±4.10 | 4.58±0.07 |
|  | AG (146) | 15.27±0.43 | 2858.89±88.12 | 225.64±8.15^a^ | 7.87±0.21 | 129.59±4.09 | 4.52±0.07 |
|  | GG (48) | 14.99±0.43 | 2727.22±88.75 | 214.58±8.21^b^ | 7.80±0.21 | 123.98±4.12 | 4.52±0.07 |
|  | P value | 0.3647 | 0.1105 | 0. 0287 | 0.3612 | 0.0702 | 0.0693 |
| g.530394C>G | CC (99) | 15.11±0.41 | 2780.47±85.85 | 219.96±7.95 | 7.87±0.20 | 126.52±4.00 | 4.54±0.07 |
|  | CG (164) | 15.38±0.42 | 2834.01±87.51 | 222.87±8.11 | 7.80±0.21 | 129.30±4.06 | 4.53±0.07 |
|  | GG (67) | 15.76±0.45 | 2907.87±93.69 | 227.71±8.68 | 7.79±0.22 | 132.98±4.35 | 4.56±0.08 |
|  | P value | 0.1782 | 0.1042 | 0. 1903 | 0.6756 | 0.0919 | 0.9293 |
| g.531913A>C | AA (13) | 15.78±0.57^a^ | 2923.29±119.74^a^ | 225.70±11.10 | 7.74±0.28 | 133.46±5.56^a^ | 4.58±0.10 |
|  | CA (93) | 14.82±0.41^b^ | 2743.01±86.22^b^ | 216.85±7.99 | 7.80±0.20 | 124.68±4.00^b^ | 4.50±0.07 |
|  | CC (225) | 15.66±0.41^a^ | 2870.43±85.87^a^ | 225.95±7.96 | 7.87±0.20 | 131.09±4.00^a^ | 4.58±0.07 |
|  | P value | 0.0173 | 0.0479 | 0.1576 | 0.3614 | 0.0395 | 0.8464 |
| g.532009C>T | CC (225) | 15.63±0.41^a^ | 2865.15±86.05 | 225.57±7.97 | 7.87±0.20 | 130.77±4.00 | 4.57±0.07 |
|  | TC (94) | 14.84±0.41^b^ | 2749.50±86.33 | 217.27±7.99 | 7.80±0.20 | 125.05±4.01 | 4.50±0.07 |
|  | TT (12) | 15.36±0.60^a^ | 2838.48±124.18 | 219.44±11.50 | 7.74±0.29 | 128.16±5.77 | 4.54±0.10 |
|  | P value | 0. 0340 | 0. 0814 | 0.1773 | 0.3936 | 0.0734 | 0.6629 |
| g.532389A>C | AA (223) | 15.62±0.41^a^ | 2864.02±86.12 | 225.33±7.98 | 7.87±0.20 | 130.62±4.00 | 4.57±0.07 |
|  | CA (97) | 14.86±0.41^b^ | 2752.92±86.26 | 217.69±8.00 | 7.81±0.20 | 125.31±4.01 | 4.51±0.07 |
|  | CC (11) | 15.38±0.61^a^ | 2841.15±126.50 | 219.94±11.72 | 7.76±0.30 | 127.90±5.88 | 4.52±0.11 |
|  | P value | 0. 0420 | 0. 0942 | 0.2109 | 0.5943 | 0.0968 | 0.5960 |
| g.534640A>G | AA (223) | 15.63±0.41^a^ | 2864.28±86.13 | 225.31±8.00 | 7.87±0.20 | 130.64±4.00 | 4.57±0.07 |
|  | GA (96) | 14.86±0.41^b^ | 2752.74±86.27 | 217.71±8.00 | 7.81±0.20 | 125.29±4.01 | 4.51±0.07 |
|  | GG (12) | 15.36±0.60^a^ | 2838.90±124.24 | 219.40±11.51 | 7.73±0.29 | 128.15±5.78 | 4.54±0.10 |
|  | P value | 0. 0442 | 0. 0963 | 0.2141 | 0.3836 | 0.0956 | 0.6856 |

Values of milk production traits in each genotypes are represented as mean±SE. Values with different superscripts within the same column in were statistically different at *P*<0.05.
